# Laparoscopic transperitoneal adrenalectomy in the large adrenal tumor from single center experience

**DOI:** 10.1186/s12893-021-01080-y

**Published:** 2021-02-01

**Authors:** Thanasit Prakobpon, Apirak Santi-ngamkun, Manint Usawachintachit, Supoj Ratchanon, Dutsadee Sowanthip, Kamol Panumatrassamee

**Affiliations:** Division of Urology, Department of Surgery, Faculty of Medicine, Chulalongkorn University, King Chulalongkorn Memorial Hospital, The Thai Red Cross Society, 1873 Rama 4 Road, Pathumwan, Bangkok, 10330 Thailand

**Keywords:** Adrenalectomy, Laparoscopy, Large adrenal tumor, Safety

## Abstract

**Background:**

The role of laparoscopic adrenalectomy (LA) in a large adrenal tumor is controversial due to the risk of malignancy and technical difficulty. In this study, we compared the perioperative outcomes and complications of LA on large (≥ 6 cm) and (< 6 cm) adrenal tumors.

**Methods:**

We retrospectively reviewed all clinical data of patients who underwent unilateral transperitoneal LA in our institution between April 2000 and June 2019. Patients were classified by tumor size into 2 groups. Patients in group 1 had tumor size < 6 cm (n = 408) and patient in group 2 had tumor size ≥ 6 cm (n = 48). Demographic data, perioperative outcomes, complications, and pathologic reports were compared between groups.

**Results:**

Patients in group 2 were significant older (*p* = 0.04), thinner (*p* = 0.001) and had lower incident of hypertension (*p* = 0.001), with a significantly higher median operative time (75 vs 120 min), estimated blood loss (20 vs 100 ml), transfusion rate (0 vs 20.8%), conversion rate (0.25 vs 14.6%) and length of postoperative stays ( 4 vs 5.5 days) than in group 2 (all *p* < 0.001). Group 2 patients also had significantly higher frequency of intraoperative complication (4.7 vs 31.3%; adjust Odds Ratio [OR] = 9.67 (95% CI 4.22–22.17), *p*-value < 0.001) and postoperative complication (5.4 vs 31.3%; adjust OR = 5.67 (95% CI 2.48–12.97), *p*-value < 0.001). Only eight (1.8%) major complications occurred in this study. The most common pathology in group 2 patient was pheochromocytoma and metastasis.

**Conclusions:**

Laparoscopic transperitoneal adrenalectomy in large adrenal tumor ≥ 6 cm is feasible but associated with significantly worse intraoperative complications, postoperative complications, and recovery. However, most of the complications were minor and could be managed conservatively. Careful patient selection with the expert surgeon in adrenal surgery is the key factor for successful laparoscopic surgery in a large adrenal tumor.

*Trial registration:* This study was retrospectively registered in the Thai Clinical Trials Registry on 02/03/2020. The registration number was TCTR20200312004.

## Background

At present, laparoscopic adrenalectomy (LA) has become the standard treatment for all small adrenal tumors since first being introduced in 1992 by Gagner et al. [1]. The superiority of LA has resulted in less postoperative pain, decreased length of hospital stay, and better cosmetic results compared to open adrenalectomy [2–7]. The only specific contraindications for LA are adrenocortical carcinoma (ACC) that have radiologic evidence of tumor invasion to the periadrenal tissue, the local recurrent tumor of a previously resected adrenal mass, and patients with severe cardiopulmonary disease [8].

The role of LA for a large adrenal tumor is still a debatable issue. There are two major problems of concern. The first is the technical difficulties in removal of a large adrenal tumor can prolong the operative time, increase the blood loss, and lead to perioperative complications. The second is that the risk of malignancy is directed related to the size of the tumor [9]. Laparoscopic surgery for adrenal cancer still has doubts over the potential of incomplete resection, tumor spillage, and capsular disruption that can cause local tumor recurrence and metastasis [10].

There is no clear definition of the size of a large adrenal tumor. However, the European Society of Endocrinology Clinical Practice Guideline recommended performing laparoscopic surgery for adrenal tumors size < 6 cm which is no local invasion. An individualized surgical approach is recommended for every adrenal tumor size > 6 cm [11].

This study aimed to evaluate the safety and feasibility of LA in patient with a large adrenal tumor by comparing the perioperative outcomes of LA between patients with small (< 6 cm) and large (≥ 6 cm) in our hospital.

## Methods

### Study populations

This study was registered in the Thai Clinical Trials Registry on 02/03/2020 (Registration number was TCTR20200312004) and the study protocol obtained approval from the Institution-Review Board from our University. The study has been reported in line with the strengthening the reporting of cohort studies in surgery (STROCSS) criteria [12].

Inclusion criteria in this study were patients > 18 years old who was performed unilateral laparoscopic transperitoneal total adrenalectomy between April 2000-June 2019 in our hospital which is a Tertiary care University hospital. All clinical data were retrospectively reviewed. Patients were divided into 2 groups based on their tumor size. Group 1 had tumor size < 6 cm and group 2 had tumor size ≥ 6 cm. Tumor size was measured at the maximum dimension based on the pathological report.

Indications for LA were functioning adrenal tumors, non-functioning adrenal tumors larger than 4 cm and enlarged adrenal tumors during follow up. Large tumor size, high patient’s comorbidities, and localized ACC which were suspected by imaging study were not a contraindication for LA in our institution, but the choice between LA or open surgery was an individualized selection based on the surgeon’s and patient’s preferences. Our contraindications for laparoscopy were a local recurrence of the previously resected adrenal tumor and a locally advanced ACC.

### Patient preparations

All patients completed a standard hormonal evaluation by the endocrinologist. Computed tomography (CT) adrenal protocol including a washout study was the first choice of diagnostic imaging. Patient contraindicated to a CT scan were examined by magnetic resonance imaging (MRI) instead. An additional CT chest was performed in patients suspected of ACC and malignant pheochromocytoma.

In functioning adrenal tumors, the blood pressure was controlled with antihypertensive medication before surgery. Serum potassium level was normalized by potassium replacement in aldosterone-producing adenoma patients. Hydrocortisone replacement was given on the day of surgery in Cushing syndrome patients.

In pheochromocytoma patients, the alpha-adrenergic blockade was titratable to control the blood pressure and heart rate at least 2 weeks before surgery. The patient was motivated to intake a high salt diet and high fluid intake. The intensive care unit was prepared for postoperative care in pheochromocytoma patients.

### Surgical techniques

All LAs were performed by a standard lateral transperitoneal approach with patient in the lateral decubitus position as previously described [13]. In brief, four trocars were used for a right adrenalectomy and three for a left adrenalectomy. An additional retracting trocar may be needed in patients with a large adrenal tumor.

For a right adrenalectomy, the right lobe of the liver was mobilized upwards. The peritoneum lateral to inferior vena cava was then opened longitudinally and the adrenal gland was dissected from the medial to the lateral direction and from the inferior to superior direction. For a left adrenalectomy, the descending colon was mobilized medially to expose the upper pole of the kidney. Then, the tail of the pancreas and spleen were rotated medially. The left adrenal vein was controlled, and the adrenal gland was dissected from the medial to the lateral direction. Lastly, the adrenal gland was dissected from the upper pole of the kidney.

Adrenal arteries and small adrenal veins were controlled using Ligasure, a vessel sealing device, Covidien-Medtronic (clipless adrenalectomy) [14]. The large adrenal vein was controlled with a Titanium clip or Hem-o-lok clip. A close-suction drain was not routinely used, but it was placed only in patients with blood oozing from the surgical bed.

### Outcomes measurement and data analysis

Analyzed parameters included age, gender, body mass index (BMI), tumor side, tumor size, comorbidities, American Society of Anesthesiologists (ASA) score, previous abdominal surgery, operative time, estimated blood loss (EBL) which was measured by laparoscopic suction device, transfusion rate, conversion rate, complications, pathological reports, margin status, and length of postoperative stay.

The primary outcome was to compare the complication rates between the two groups and the secondary outcome was to compare the perioperative parameters and length of postoperative stay. The 30-day postoperative complication was graded according to the Clavien-Dindo Classification [15]. Grade 1–2 complication was defined as a minor complication and grade 3–5 complication was defined as a major complication. Patients in group 2 were subgroup to pheochromocytoma and non-pheochromocytoma group, perioperative parameters were compared between groups.

Categorical data were analyzed using Fisher's exact test or Chi-square test and reported as a number (percentage). Continuous data were analyzed using an independent t-test or Mann Whitney test and reported as the median and interquartile range (IQR). Logistic regression analyses were performed to determine risk factors associated with intraoperative and postoperative complications by using the patient’s demographic data including the diagnosis of pheochromocytoma. Variables with p-value < 0.1 in univariable analysis were selected in a multivariable logistic regression model. Statistical analysis was performed using STATA version 15, accepting P-value < 0.05 as statistically significant.

## Results

In total, 527 patients underwent LA in our institution during April 2000-June 2019. From the selection criteria, 456 patients were included in this study and were comprised of 408 patients in group 1 (tumor < 6 cm) and 48 patients in group 2 (tumor ≥ 6 cm). Baseline patient’s characteristics are presented in Table 1. Patient in group 2 were significantly older (47.4 vs 51.4, *p* = 0.04) and thinner (BMI = 25.6 vs 23.2, p = 0.001) than patients in group 1. Preoperative diagnosis was significantly different between groups (*p* < 0.001). Group 1 patients had a higher incidence of hypertension (89.5% vs 72.9%, *p* = 0.001) and higher ASA score (*p* 0.001) than group 2 patients. The largest tumor size in this study was 17 cm.

**Table 1:** Baseline patient’s characteristics

	Group 1 (N = 408) Tumor size < 6 cm	Group 2 (N = 48) Tumor size ≥ 6 cm	*P*-value
Age, years, median (IQR)	47.4 (38–56)	51.4 (43–60)	0.04*
Gender, male: female, N (%)	139 (34.1): 269 (65.9)	15 (31.3): 33 (68.7)	0.70
BMI, kg/^m2^, median (IQR)	25.6 (22.8–28.4)	23.2 (19.6–26.5)	0.001*
Tumor laterality, right: left, N (%)	179 (43.9): 229 (56.1)	26 (54.2): 22 (45.8)	0.18
Tumor size, cm, median (IQR)	2 (1.5–3)	8 (6.6–10)	< 0.001*
Comorbidities, N (%)			
Diabetes	77 (18.9)	11 (22.9)	0.50
Hypertension	365 (89.5)	35 (72.9)	0.001*
Chronic kidney disease	21 (5.2)	2 (4.2)	0.77
Dyslipidemia	74 (18.1)	10 (20.8)	0.65
ASA classification, N (%)			< 0.001*
1	21 (5.2)	11 (22.9)	
2	357 (87.5)	35 (72.9)	
3	30 (7.4)	2 (4.2)	
Previous abdominal surgery, N (%)	136 (33.8)	15 (32.6)	0.87
Preoperative diagnosis, N (%)			< 0.001*
APA	267 (65.4)	2 (4.2)	
Cushing syndrome	59 (14.5)	2 (4.2)	
Incidentaloma	34 (8.3)	16 (33.3)	
Pheochromocytoma	36 (8.8)	21 (43.8)	
Metastasis	7 (1.7)	4 (8.3)	
Myelolipoma	1 (0.2)	2 (4.2)	
Adrenocortical carcinoma	4 (1.2)	1 (2.1)	

*IQR* interquartile range, *BMI *body mass index, *ASA* American Society of Anesthesiologists,** APA** aldosterone-producing adenoma

*P-value < 0.05

Perioperative outcomes are shown in Table 2. Operative time (75 vs 120 min), EBL (20 vs 100 ml) and transfusion rate (0 vs 20.8%) were significant higher in group 2 patient (*p* < 0.001). Nineteen (4.7%) and fifteen (31.5%) intraoperative complications occurred in group 1 and group 2 respectively (*p* < 0.001). The most common intraoperative complications in group 1 were ten minor liver injuries, which were treated with electrocautery, followed by two serosa tears of the colon that were repaired with laparoscopic suturing. Intraoperative complications in group 2 were five tumor capsular tears, four massive bleedings, three splenic injuries which required laparoscopic splenectomy in one patient, one liver injury, one inferior vena cava injury, and one diaphragmatic injury.

**Table 2:** Perioperative outcomes

	Group 1 (N = 408) Tumor size < 6 cm	Group 2 (N = 48) Tumor size ≥ 6 cm	*P*-value
Intraoperative parameters			
Operative time, minutes, median (IQR)	75 (60–108)	120 (100–150)	< 0.001*
Estimated blood loss, ml, median (IQR)	20 (20–50)	100 (30–300)	< 0.001*
Transfusion, N (%)	0	10 (20.8)	< 0.001*
Intraoperative complication, N (%)	19 (4.7)	15 (31.3)	< 0.001*
Open conversion, N (%)	1 (0.25)	7 (14.6)	< 0.001*
Postoperative parameters			
30 day postoperative complication, N (%)	22 (5.4)	15 (31.3)	< 0.001*
Minor (Clavien grade 1–2), N (%)	17 (4.2)	12 (25)	< 0.001*
Major (Clavien grade 3–5), N (%)	5 (1.2)	3 (6.2)	0.04*
Positive surgical margin, N (%)	5 (1.2)	2 (4.2)	0.16
Length of postoperative stay, day, median (IQR)	4 (4–5)	5.5 (5–7)	< 0.001*

*IQR *interquartile range

*P-value < 0.05

Eight open conversions occurred in this study, comprised of one patient (0.25%) in group 1 and seven patients (14.6%) in group 2 (*p* < 0.001). The causes of open conversion were massive bleeding (one patient in group 1 and five patients in group 2), liver injury (one patient), and inferior vena cava injury (one patient). Length of postoperative hospital stay was significantly longer in group 2 patient (4 vs 5.5 days, *p* < 0.001).

The overall 30-day postoperative complications (5.4 vs 31.3%, *p* < 0.001), minor postoperative complications (4.2 vs 25%, *p* < 0.001) and major postoperative complications (1.2 vs 6.2%, *p* = 0.04) were significant higher in group 2.

Grades of postoperative complications according to the Clavien-Dindo classification are presented in Table 3. The most common complications were transfusion and infection of the wound. There were eight major complications in this study. In group 1, there was one postoperative bleeding that required explore laparotomy, one incisional hernia treated with surgical repair, one acute renal failure, and two pulmonary embolisms. In group 2, there were two pleural effusions which were treated with needle aspiration, and one 9.5-cm pheochromocytoma patient had an ischemic stroke with hemiparesis after the operation.

**Table 3:** Details of postoperative complications

Clavien-Dindo Classification	Group 1 (N = 408) Tumor size < 6 cm	Group 2 (N = 48) Tumor size ≥ 6 cm
Grade 1	Atelectasis (2), subcutaneous emphysema at scrotum (2), prolonged bowel ileus (1), pyelonephritis (1), phlebitis (1), desaturation (1)	Pleural effusion (1), partial gut obstruction (1)
Grade 2	Infected wound (4), congestive heart failure (2), pleural effusion (1), ventricular tachycardia (1), vertebral artery dissection (1)	Transfusion (10)
Grade 3	Reoperation (1), incisional hernia (1)	Pleural effusion (2)
Grade 4	Pulmonary embolism (2), acute renal failure (1)	Ischemic stroke (1)
Grade 5	None	None

The pathological reports are summarized in Table 4. Most of the patients with a small adrenal tumor was cortical adenoma (75.3%). In contrast, the most common pathology in patients with a large adrenal tumor group were pheochromocytoma (41.7%) and metastasis (10.4%). The incidence of ACC in group 2 (8.3%) was higher than in group 1 (2.2%).

**Table 4:** Pathological reports

Pathology	Group 1 (N = 408) Tumor size < 6 cm	Group 2 (N = 48) Tumor size ≥ 6 cm
Cortical adenoma	307 (75.3)	2 (4.2)
Nodular hyperplasia	37 (9.1)	1 (2.1)
Pheochromocytoma Benign Malignant	25 (6.1) 19 (4.6) 6 (1.5)	20 (41.7) 13 (27) 7 (15)
Adrenocortical carcinoma	9 (2.2)	4 (8.3)
Myelolipoma	7 (1.7)	4 (8.3)
Metastasis	6 (1.5)	5 (10.4)
Schwannoma	3 (0.7)	4 (8.3)
Other	14 (3.4)	8 (16.7)

Report as number (percentage)

We subgroup analyzed the patients in group 2 by comparing the perioperative outcomes between pheochromocytoma (N = 20) and non-pheochromocytoma (N = 28) patients, with the results shown in Table 5. There was no statistical difference between the two groups in terms of operative time, EBL, transfusion rate, conversion rate, complications, surgical margin status, and length of postoperative stay.

**Table 5:** Subgroup analysis perioperative outcomes of patient in group 2 (tumor ≥ 6 cm)

	Non-pheochromocytoma (N = 28)	Pheochromocytoma (N = 20)	*P*-value
Intraoperative parameters			
Operative time, minutes, median (IQR)	120 (100–150)	132 (100–155)	0.46
Estimated blood loss, ml, median (IQR)	100 (20–300)	250 (50–425)	0.14
Transfusion, N (%)	3 (11.5)	7 (35)	0.08
Intraoperative complication, N (%)	9 (32.1)	6 (30)	0.87
Open conversion, N (%)	4 (14.3)	3 (15)	0.94
Postoperative parameters			
30 day postoperative complication, N (%)	6 (21.4)	9 (45)	0.12
Minor (Clavien grade 1–2), N (%)	5 (17.9)	7 (35)	0.20
Major (Clavien grade 3–5), N (%)	1 (3.6)	2 (10)	0.56
Positive surgical margin, N (%)	2 (7.1)	0	0.50
Length of postoperative stay, day, median (IQR)	5 (4–7)	6 (5–7.5)	0.24

*IQR* interquartile range

Logistic regression analysis to determine risk factors associated with intraoperative and postoperative complications were presented in Table 6. Tumor size ≥ 6 cm represented the only significant preoperative parameter predictive of intraoperative and postoperative complication in both univariable and multivariable analysis (all *p* value < 0.001). Diagnostic of pheochromocytoma was a significant predictive factor of postoperative complication in univariable analysis (*p* < 0.001). However, this was not confirmed to be predictive in the multivariable model (*p* = 0.06).

**Table 6:** Logistic regression analysis between preoperative parameters and complications

	Intraoperative complication				Postoperative complication			
	Univariate		Multivariate		Univariate		Multivariate	
	OR (95%CI)	*p*-value	aOR (95% CI)	*p*-value	OR (95% CI)	*p*-value	aOR (95% CI)	*p*-value
Age ≥ 50 years	1.8 (0.9–3.58)	0.09	1.46 (0.7–3.05)	0.32	1.99 (1.01–3.92)	0.05	1.53 (0.74–3.18)	0.26
BMI ≥ 25 kg/m^2^	0.67 (0.34–1.34)	0.67	–	–	1.09 (0.56–2.12)	0.8	–	–
Tumor size ≥ 6 cm	9.3 (4.33–20)	< 0.001*	9.67 (4.22–22.17)	< 0.001*	6.59 (3.12–13.9)	< 0.001*	5.67 (2.48–12.97)	< 0.001*
Comorbidities								
Diabetes	1.21 (0.53–2.76)	0.64	–	–	1.33 (0.61–2.93)	0.48	–	–
Hypertension	0.55 (0.23–1.32)	0.18	–	–	0.72 (0.29–1.82)	0.49	–	–
Chronic kidney disease	2.64 (0.85–8.22)	0.09	3.13 (0.92–10.63)	0.07	2.47 (0.8–7.68)	0.12	–	–
Dyslipidemia	1.08 (0.45–2.54)	0.87	–	–	1.42 (0.65–3.12)	0.38	–	–
ASA classification		0.35	–			0.65	–	–
1	Ref				Ref			
2	0.42 (0.15–1.16)				0.62 (0.21–1.88)			
3	0.56 (0.12–2.57)				0.47 (0.08–2.75)			
Previous abdominal surgery	0.98 (0.48–2.02)	0.96	–	–	1.02 (0.51–2.07)	0.95	–	–
Pheochromocytoma	2.27 (0.98–5.26)	0.06	1.02 (0.38–2.73)	0.97	3.46 (1.61–7.46)	< 0.001*	2.28 (0.97–5.4)	0.06

Variables with *p* value < 0.1 in univariable analysis were selected in a multivariable logistic regression model

*BMI*  body mass index, *ASA* American Society of Anesthesiologists

*P-value < 0.05

## Discussion

Currently, LA is the first surgical treatment of choice for almost all adrenal tumors since it provides better perioperative outcomes compared to traditional open adrenalectomy [2–7]. However, LA had limitations in some groups of patients, including patients with a large adrenal tumor. Nowadays, there is no cut-off point of tumor size for contraindication in the laparoscopic approach. Technical difficulties from highly vascularized supply and possible involvement of the surrounding organs may worsen the perioperative outcomes [16]. Nevertheless, the previous literatures have demonstrated the success of using LA for removal of large adrenal tumor [17–22].

Hemal et al. [17] reported the outcomes of LA for an adrenal tumor size of > 5 cm in 22 patients from both transperitoneal and retroperitoneal approaches, and concluded that the tumor size was not the primary factor to consider using open surgery rather than LA. Agrusa et al. [18] presented the outcomes of LA in 14 patients with a large adrenal tumor size of > 6 cm. (mean 8.2 cm) and found no capsular disruption, postoperative mortality, and open conversion occurred. Moreover, Parnaby et al. [19] compared the outcomes of LA for adrenal tumors of ≥ 6 cm with those of < 6 cm in 101 patients. They concluded in the absence of local invasion; the perioperative outcomes were comparable between the two groups. On the other hand, Natkaniec et al. [16] reported a large comparative study of 530 patients and found a significantly higher operative time, EBL, and conversion rate when using LA for adrenal tumor sizes of ≥ 6 cm.

For larger tumors, Zografos et al. [20] evaluated the outcomes of LA for adrenal tumors of ≥ 8 cm in 15 patients and summarized that laparoscopic resection was safe and feasible. Bozkurt et al. [21] compared the outcomes of transperitoneal LA between adrenal tumors of ≥ 8 cm (n = 16) and < 8 cm (n = 19) and found the operative time and EBL were higher in the large adrenal tumor group, but the difference did not reach a significant level (*p* = 0.05).

Moreover, LA has also shown a good feasibility for huge benign adrenal tumors. Abraham et al. [22] reported a case of a 17 cm adrenal ganglioneuroma that was successfully removed by LA while Bozkurt et al. [21] reported a 15 cm myelolipoma treated with LA. In our study, the largest tumor was a 17 cm mature cystic teratoma.

For the surgical approach, transperitoneal LA in the lateral decubitus position is the most popular technique among surgeons. This technique provides a large working space, and safe for both small and large adrenal tumors [16, 18–21, 23, 24]. Retroperitoneoscopic adrenalectomy has the advantages of direct access to the adrenal and avoids entering the peritoneal cavity. Wang et al. [25] demonstrated the safety and feasibility of retroperitoneoscopic adrenalectomy that performed by experienced surgeons in 110 patients with a large adrenal tumor size of > 5 cm (mean tumor size was 7.2 cm). The American Society for Gastrointestinal and Endoscopic Surgery recommends surgeon to choose the approach which is the most familiar. The transperitoneal approach is particularly interesting in a large adrenal tumor size of > 6 cm and in morbid obesity. Conversely, the retroperitoneal approach is associated with a lower complication and shorter operative time in the patient with previous abdominal surgery [26].

The risk of malignancy in an adrenal tumor is directly correlated with the tumor size, where the role of laparoscopic surgery in ACC is still questionable. The incidence of ACC in adrenal incidentaloma is 1% for tumors of < 4 cm, 6% for tumors of 4–6 cm and 20% for tumors of > 6 cm [9]. Although ACC is rare, it is highly aggressive tumor. Therefore, benefits of minimally invasive surgery from laparoscopic surgery must be weighed with the risk of incomplete resection and capsular perforation which can worsen the oncological outcomes.

Although some authors have suggested avoiding LA for adrenal tumors that are suspected of ACC and of > 6 cm [27], some authors have shown the safe and feasible use of LA in ACC. Brix et al. [28] compared the oncologic outcomes in 152 patients with ACC size of ≤ 10 cm between open adrenalectomy and LA using matched-pairs analysis. They found no statistical difference in disease-specific survival, recurrence-free survival, frequency of tumor capsule violation, and postoperative peritoneal carcinomatosis between two types of surgery. Machado et al. [29] reported a systematic review in the role of LA in ACC and found no difference in the oncologic outcomes between open and laparoscopic approaches. They concluded that a poor surgical outcome is more likely from an inadequate surgery than the surgical approach. In contrast, Wu et al. [10] found non-equivalent oncologic outcomes, with a higher recurrent rate and shorter time to recurrence in LA compared to open adrenalectomy. Cooper et al. [30] also reported a higher peritoneal recurrence, shorter recurrence-free survival, and lower overall survival from the use of LA in ACC compared with an open surgery approach.

Robot-assisted surgery has been widely accepted as a standard minimally invasive surgical treatment in various kinds of surgery. However, the role of robotic adrenalectomy (RA) is still debatable. RA seems to be safe and feasible than LA, but there is currently no large prospective controlled trial that showed the superiority of RA over LA. Economopoulos et al. [31] presented a large meta-analysis in 1162 patients and concluded that RA was associated with longer operative time than LA. Intraoperative complications, postoperative complications, mortality rates and conversion rates were not significantly different between RA and LA.

For the specific patients with adrenal tumors of ≥ 6 cm, Morelli et al. [32] showed a shorter operative time in RA and concluded RA had potential benefits over LA in patient with adrenal tumors of ≥ 6 cm, BMI ≥ 30 kg/m^2^, and patient with previous abdominal surgery.

In this study, we reported the surgical outcomes in LA from our 20-year experiences in our high-volume referral center. This study included large number of patients with a wide range of preoperative diagnosis. Some of the patient’s characteristics were different because of the different kinds of patients between groups. Patient in group 2 was significantly thinner, which may be due to the high proportion of pheochromocytoma patients. A lower incidence of hypertension and ASA score in group 2 could be from the higher proportion of asymptomatic non-functioning adrenal incidentaloma in group 2 patients and a higher proportion of functioning adrenal tumor in group 1 patients.

Our results revealed significantly worse perioperative outcomes in patients with a tumor size of ≥ 6 cm, except the positive margin status was not significantly different between the two groups. Logistic regression analysis also showed a significant increase in the intraoperative and postoperative complications in adrenal tumors of ≥ 6 cm from both unadjusted and adjusted ORs. Pheochromocytoma was associated with increase postoperative complication in the univariable analysis but failed to be a predictor in multivariable analysis. However, the most common postoperative complication in group 2 was transfusion. Moreover, only three major complications occurred in group 2, and all were medical complications.

There was a high proportion of pheochromocytoma in group 2 patients. We subgroup analysis of the perioperative outcomes between patients with and without pheochromocytoma revealed that the rates of transfusion (35 vs 11.5%) and postoperative complication (45% vs 21.4%) were numerically but not significantly higher in the pheochromocytoma group (*p* = 0.08 and 0.12, respectively).

The incidence of ACC was higher in group 2 patients (8.3% vs 2.2%). During the follow-up period, two patients (22.2%) in group 1 and one patient (25%) in group 2 death because of tumor progression and metastasis. Although the survival rates of ACC patients didn’t seem to differ between the 2 groups. We were unable to compare the oncological results from this study because there were a very small number of ACC patients.

Overall, we believe that LA of large adrenal tumors is safe and feasible for a suitably experienced surgeon. For tumors that are suspected of adrenal cancer, oncologic principles must be strictly followed with minimal touch technique, en-bloc tumor resection with a wide surgical margin, preserved an intact tumor capsule, and a low threshold of open conversion so as to obtain the appropriate surgical margin and oncological results.

The limitation of this study was a non-randomized retrospective design. The number of patients between 2 groups is quite different. The LAs in this study were performed by multiple surgeons and there may have been a selection bias for choosing the surgical approach in large adrenal tumors among surgeons. Our findings should be counseled to patients before performing LA in a large adrenal tumor. A prospective randomized controlled trial is needed for future study.

## Conclusions

Laparoscopic transperitoneal adrenalectomy in large adrenal tumor ≥ 6 cm is feasible but associated with significantly worse intraoperative complications, postoperative complications and recovery. However, most of the complications were minor and could be managed conservatively. Careful patient selection with the expertise surgeon in adrenal surgery are the key factors for successful laparoscopic surgery in a large adrenal tumor.

## Data Availability

The datasets used and/or analyzed during the current study are available from the corresponding author on reasonable request.

## Abbreviations

LA: Laparoscopic adrenalectomy
ACC: Adrenocortical carcinoma
CT: Computed tomography
MRI: Magnetic resonance imaging
BMI: Body mass index
ASA: American Society of Anesthesiologists
EBL: Estimated blood loss
IQR: Interquartile range
OR: Odd ratio
CI: Confidence interval
RA: Robotic adrenalectomy
